# Endoscopic direct therapy for appendicitis and diverticulitis in one patient with right-sided abdominal pain

**DOI:** 10.1055/a-2361-1361

**Published:** 2024-08-07

**Authors:** Jianzhen Ren, Silin Huang, Jun Cai, Bo Li, Guang Yang, Suhuan Liao, Ronggang Zhang

**Affiliations:** 1 Department of Gastroenterology, South China Hospital, Medical School, Shenzhen University, Shenzhen, China

Acute appendicitis and cecal diverticulitis are both common causes of acute right-sided abdominal pain, but it is extremely rare for both to be found in one patient. Acute appendicitis and diverticulitis are mainly treated through medication and surgical intervention 1 2 . Digital single-operator cholangioscopy (dSOC) has proven effective for managing inflammation in natural conduits such as the bile duct, pancreatic duct, and appendix 3 4 . Herein, we present endoscopic direct therapy for appendicitis and diverticulitis in a man with right-sided abdominal pain ( Video 1 ).

Initial report on the application of digital single-operator cholangioscopy for endoscopic direct therapy.Video 1

A 33-year-old man presented with right-sided abdominal pain for 4 days. Abdominal ultrasonography showed a tubular mass in the right lower quadrant of the abdomen (3.67 × 0.81 cm) and appendicitis was considered ( Fig. 1 **a** ). Colonoscopy revealed appendicitis and cecal diverticulitis ( Fig. 1 **b** ). Endoscopic direct appendicitis therapy and endoscopic direct diverticulitis therapy utilizing dSOC was performed, and a milk-like pus was observed pouring out and a substantial volume of fecaliths ( Fig. 2 **a–d** ). These fecaliths were meticulously fragmented, extracted, and removed using a disposable basket following repeated lavages with metronidazole and sodium chloride ( Fig. 2 **e, f** ), rendering the mucosa cleansed yet characterized by roughness and swelling without evidence of perforation ( Fig. 3 **a, b** ). A 7 Fr pancreatic duct stent was strategically placed to ensure unobstructed drainage ( Fig. 4 **a, b** ). The procedure was completed in 165 minutes. The patient’s abdominal pain was relieved immediately after the procedure. Subsequent computed tomography revealed fecaliths had been removed completely and the stent discharged ( Fig. 5 ). No recurrence or adverse events were noted during the 4-month follow-up.

**Fig. 1:**
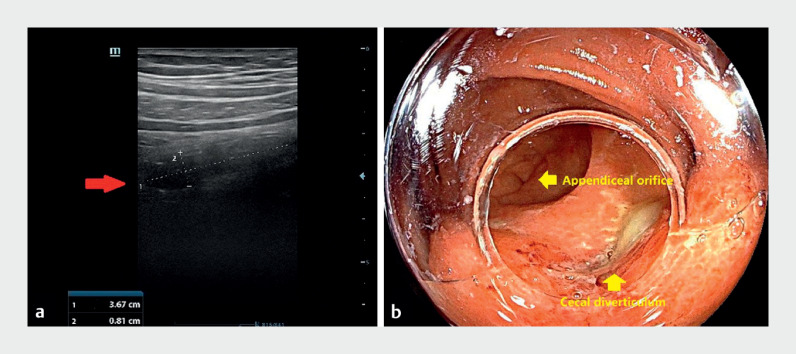
**a** Abdominal ultrasonography showed a tubular mass in the right lower quadrant of the abdomen (3.67 × 0.81 cm), and appendicitis was considered. **b** Colonoscopy revealed appendicitis and cecal diverticulitis.

**Fig. 2:**
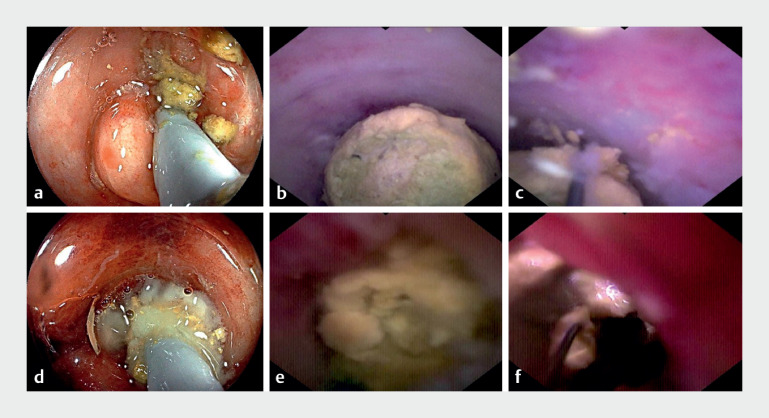
The process of endoscopic direct therapy. **a, b** A lot of milk-like pus was observed pouring out from the appendiceal orifice/cecal diverticulum. **c, d** A considerable amount of fecaliths within the diverticular cavity/appendiceal lumen was visualized using digital single-operator cholangioscopy (dSOC). **e, f** The fecaliths were dissected, extracted, and removed using a disposable basket under the visual guidance of dSOC.

**Fig. 3:**
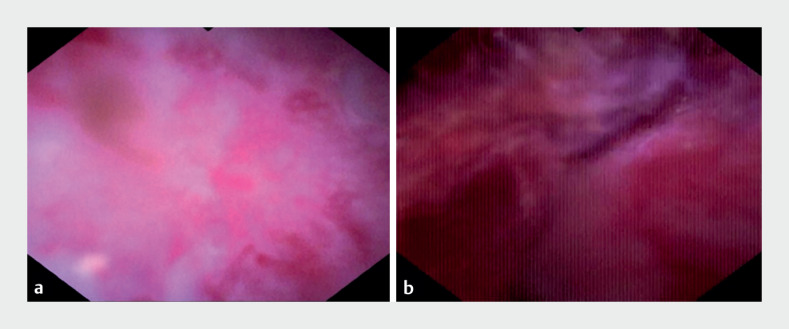
**a, b** Through endoscopic direct therapy, the diverticular cavity/appendiceal lumen was observed to be clear.

**Fig. 4:**
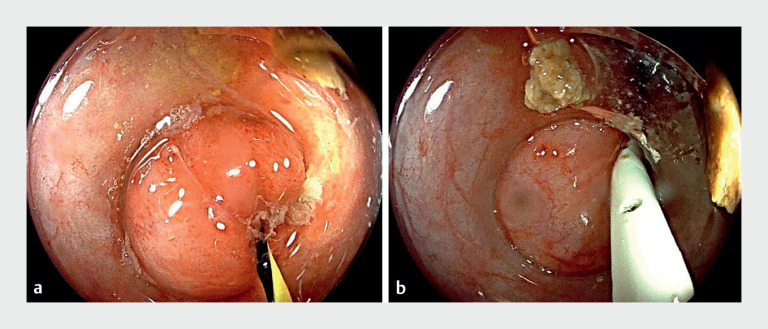
**a, b** The stent was strategically placed to ensure unobstructed drainage.

**Fig. 5:**
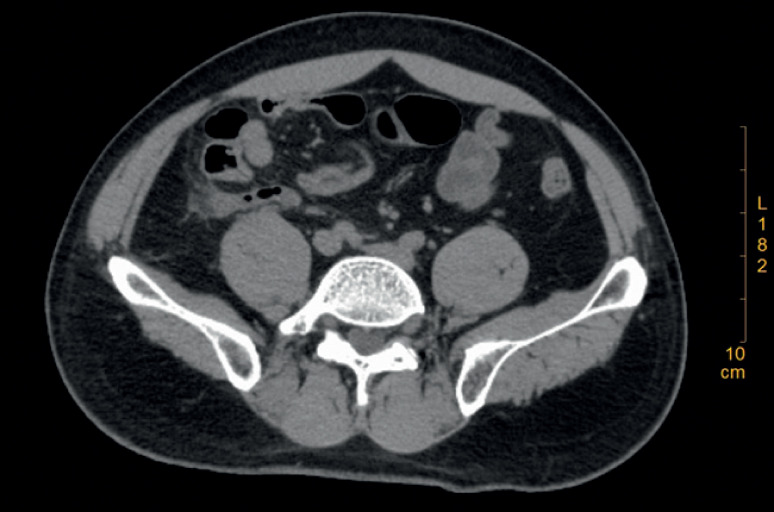
Postoperative computed tomography demonstrated fecaliths had been removed completely and the stent discharged.

As advancements in dSOC continue to evolve, significant innovations in the diagnosis and management of gastrointestinal diseases have been achieved through endoscopic direct therapy. This therapy provided a feasible, safe, and effective alternative approach for diagnosis and management of acute right-sided abdominal pain. To the best of our knowledge, this is the first reported case of a successful cure of acute appendicitis combined with diverticulitis with fecalith using endoscopic direct therapy. This combined approach could reshape the management of acute right-sided abdominal pain, emphasizing the importance of technological integration in endoscopic practices.

Endoscopy_UCTN_Code_TTT_1AQ_2AF

## Notes

Endoscopy E-Videos https://eref.thieme.de/e-videos
*E-Videos* is an open access online section of the journal *Endoscopy* , reporting on interesting cases and new techniques in gastroenterological endoscopy. All papers include a high-quality video and are published with a Creative Commons CC-BY license. Endoscopy E-Videos qualify for HINARI discounts and waivers and eligibility is automatically checked during the submission process. We grant 100% waivers to articles whose corresponding authors are based in Group A countries and 50% waivers to those who are based in Group B countries as classified by Research4Life (see: https://www.research4life.org/access/eligibility/ ). This section has its own submission website at https://mc.manuscriptcentral.com/e-videos .
